# Non-celiac Enteropathies

**DOI:** 10.1007/s11894-025-00979-3

**Published:** 2025-04-14

**Authors:** Kaitlyn Mi, Scarlett Cao, Dawn Adams

**Affiliations:** 1 Vanderbilt University School of Medicine, Nashville, TN USA; 2 Gastroenterology, Hepatology, and Nutrition, Vanderbilt University Medical Center, Nashville, TN USA

**Keywords:** Small intestine, Malabsorption, Celiac disease

## Abstract

**Purpose of Review:**

Non-celiac enteropathies (NCE) can be due to a variety of causes. The workup for NCE includes history, physical, laboratory and histology review and can be difficult. Enteropathies can result in serious illness due to consequences of malabsorption including severe weight loss, nutritional deficiencies, and debilitating diarrhea. Recognition and support of these consequences while investigating underlying etiology is essential.

**Recent Findings:**

Recent studies in NCEs have focused on improving diagnostic accuracy and predicting long-term outcomes in patients with NCEs. Further, literature has emphasized the importance of histological analysis, with a focus on differentiating between various enteropathies that cause villous atrophy, highlighting the complexity and need for personalized approaches in managing these conditions.

**Summary:**

Identification of etiologies of NCEs requires review of patients’ detailed history, medications, and lab results. Common etiologies include immunodeficiencies, infectious, iatrogenic, and malignant causes. Using a systematic approach can lead to proper diagnosis and tailor treatment choices, benefiting patient outcomes. Supportive nutrition care should be initiated early when applicable to minimize morbidity.

## Introduction

Non-celiac enteropathies (NCE) are characterized by villous atrophy without evidence of Celiac disease (CeD). This is typically defined as negative celiac serology, an insignificant or no response to a gluten-free diet (GFD) and when present, lack of celiac permissive HLA genotype (DQ2/DQ8) [1–3]. Clinically, patients with NCE present with abdominal pain, persistent diarrhea, and weight loss. Current data suggests that patients with NCEs tend to present at an older age than patients with CeD [1, 4]. Patients with NCEs also experience higher rates of mortality, in part due to age, but also because of advanced disease courses. Because of this, identification of the cause of enteropathy and prompt treatment and nutrition support is essential.

**Fig. 1:**
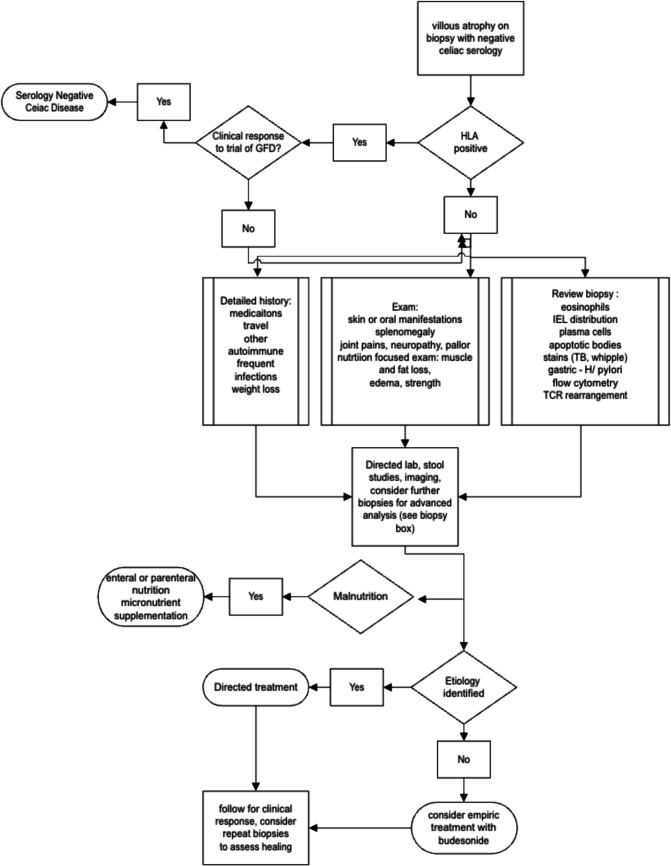
Diagnostic flowchart for NCEs

Enteropathy requires a biopsy with histology showing villous atrophy. It is important to note the pitfalls of biopsy in both under and over diagnosing atrophy. Many forms of villous atrophy can be patchy, and therefore, at minimum 4 bites should be taken of the second portion of the duodenum and 2 from the bulb with targeting mucosal abnormalities when present [5]. Although these specific numbers are based upon sensitivities for diagnosing CeD, the message of taking sufficient and targeted sampling should apply to all small bowel diseases. Many experts in the field recommend taking 1 bite per pass of forceps to improve orientation and reduce crush artifact**.**[6] After villous atrophy has been identified, other findings in the biopsy can be the clue to diagnosis. Discussion with a pathologist regarding clinical concerns and findings may be extremely productive. For example, distribution and number of intraepithelial lymphocytes, apoptosis, plasma cells, neutrophil presence, eosinophils and collagen layer abnormalities when noted may provide the diagnosis [7]. In addition to biopsy review, a detailed history and exam, including accurate review of medications and travel exposure may provide further clues to etiology [3]. An overview of the possible etiologies of NCEs are found in Table 1. A diagnostic flowchart for NCEs is found in Fig. 1.

**Table 1:** Causes of Villous Atrophy

Category	Diagnosis	Associated Findings/history	Lab Findings	Treatment	Prognosis
Known antigen trigger/autoimmune	Celiac Disease	dermatitis herpetiformis, other autoimmune conditions (hypothyroidism, type 1 diabetes, SLE, PSC/PBC), IgA deficiency	Positive TTG, EMA, crypt hyperplasia, intraepithelial lymphocytes (tip),	Gluten-free diet	Majority complete response, rare refractory disease
Autoimmune	Crohn’s Disease	Mouth sores, uveitis, arthritis, fistula, abscess, stricture other GI involvement	Elevated ESR, CRP levels, characteristic biopsy (granulomas)	Immunosuppression, steroids, immunomodulators, biologic agents	Improved with biologic therapy
	AIE	Other autoimmune diseases	Anti-enterocyte and anti-goblet cell antibody, crypt apoptosis, loss of goblet cells, associated gastritis/colitis	Immunosuppression, tacrolimus, budesonide/steroids, biologics	Variable pending response to steroid sparing drugs
	Collagenous Sprue	Older female, possible olmesartan use	Thick subepithelial collagen band	Immunosuppression, budesonide/steroids, stop olmesartan (if applicable) biologics	Poor (unless due to olmesartan)
Immunodeficiency/Allergy-like	CVID	Known, CVID, recurrent respiratory infections, low serum total protein	Low Ig, lack of plasma cells, apoptosis	Steroids, biologics	Poor
	Eosinophilic	Other atopic disorder, allergies	Biopsy and serum eosinophilia, high IgE	Elimination diet, steroids, mast cell stabilizers, leukotriene inhibitors, Dupilumab	Had been poor, improving with new therapies
Infectious	Tropical Sprue	Typically associated with tropical residence or recent travel	Low folate and/or vitamin B12	tetracycline and oral folic acid	Typically resolves post-treatment, can recur with reinfection
	Giardiasis	Steatorrhea (classically), chronic infection with IgA deficiency	Stool giardia antigen, O&P, organisms on biopsy	Metronidazole	self-resolving, chronic infection with IgA deficiency
	Whipple’s Disease	Peripheral lymphadenopathy, peripheral edema, pericarditis or endocarditis, hyperpigmentation, central nervous system symptoms	PAS-positive foamy macrophages in the small bowel biopsy	Ceftriaxone or penicillin and bactrim	Untreated can be fatal, risk of relapse
	H. Pylori	Dyspepsia, abdominal pain, nausea, and vomiting, duodenal and gastric ulcers	Positive stool Ag, gastric biopsy, urea breath test	Quadruple therapy: bismuth, metronidazole, tetracycline, and a proton pump inhibitor	H. Pylori increases risk of gastric cancers
	Tuberculosis	Constitutional symptoms (fever, malaise, night sweats, anorexia, and weight loss)	Biopsies with caseating granulomas, positive mycobacterial culture/ NAAT	Rifampin, isoniazid, pyrazinamide, ethambutol	May require surgical intervention for complications
	Viral	Acute onset, may be associated with vomiting	GI pathogen panel (if immunocompromised)	Supportive (oral rehydration solution)	Typically self-resolving, prolonged in immunocompromised or CVID
	HIV	Diarrhea, weight loss, other HIV-associated infections	Positive HIV viral titer, Villous atrophy, crypt hyperplasia, villous blunting, and lymphocytic infiltrate	HAART	HAART may benefit some patients
	SIBO	Bloating, flatulence, abdominal pain; history of gastric bypass, hypothyroid, diabetes, ileocecal resection, IgA deficiency	Positive hydrogen breath test, culture of jejunal aspirate	Rifaximin, may require neomycin and/or other broad spectrum antibiotics	May recur
Iatrogenic	Medication	Culprit medication use (eg olmesartan, mycophenolate, checkpoint inhibitor, CAR-T)	Biopsies similar to celiac, serology negative	Removal of medication	Excellent with medication removal (with exception of CAR-T therapy)
	Radiation	History of radiation, abdominal pain, partial obstructive symptoms	Vascular fibrosis, sclerosis, leukocyte infiltration, ulcerations, crypt abscesses	Symptomatic treatment, surgery	Poor
Malignant	Enteropathy-associated T-cell lymphoma	Severe diarrhea, profound weight loss	flow cytometry, abnormal CT/PET, tumor invasion on lymph node or bowel resection	Chemotherapy, stem cell transplant, surgery	Poor

*TTG* Tissue transglutaminase, *EMA* endomysial antibody, *GFD* Gluten-free diet, *GI* gastrointestinal, *ESR* erythrocyte sedimentation rate, *CRP* C-reactive protein, *AIE* autoimmune enteropathy, *CVID* Common variable immune deficiency, *IVIG* intravenous immunoglobulin, *O&P* ova and parasite, *NAAT* nucleic acid amplification test, *HIV* human immunodeficiency virus, *HAART* highly active antiviral therapy, *SIBO* short intestinal bacterial overgrowth

The focus of this review will be etiologies of NCE, which at times can be challenging. Although most curative treatment is specific to diagnosis, supportive treatment is generalizable and is essential to reducing morbidity and mortality. Because this is a disease of the small intestine, our primary organ for absorption of micro- and macro-nutrients, most of these conditions can lead to severe malnutrition, dehydration and irreversible consequences of nutrient deficiencies if untreated. Recognition of these complications is key to disease management and should include nutrition support. This may be in the form or oral supplementation, enteral nutrition or in severe cases parenteral nutrition. Clinicians caring for NCE must recognize early malnutrition and start appropriate interventions in conjunction with dietitians.

### Autoimmune

Autoimmune enteropathy (AIE) is a rare immune-mediated disease in adults, that is hypothesized to be related to an autoimmune reaction from CD4 + T-cells and presents with chronic diarrhea, malabsorption, and weight loss [8]. AIE can involve any portion of the small bowel, and diagnosis is made in patients with chronic diarrhea, villous atrophy, non-response to GFD, lack of immunodeficiency or other source, and occasionally presence of autoantibodies. Histology may show apoptotic bodies and lymphocytic infiltrate more prominent in the crypt epithelium [9, 10]. CD4/CD8 + T cells and macrophages are found in the mucosa, along with a loss of Paneth and goblet cells; importantly, plasma cells should be present, unlike in CVID [9, 10].

AIE may manifest as a part of more systemic autoimmune diseases including IPEX (immune dysregulation, polyendocrinopathy, enteropathy, X-linked) or APECED (autoimmune phenomena polyendocrinopathy candidiasis and ectodermal dystrophy syndrome), both of which are linked to T cell dysfunction [8, 11]. Clinically, patients can develop significant malnutrition due to lack of absorptive capabilities which may necessitate use of total parenteral nutrition.^10^

There is limited data available for treatment. Typically, initial treatment involves use of corticosteroids such as budesonide or prednisone [9, 10]. Other immunosuppressive agents have been described as well including 6-MP, azathioprine, tacrolimus, cyclosporine, and even biologic agents such as rituximab and infliximab [9, 12, 13].

### Crohn’s Disease

Crohn’s disease can commonly manifest with small bowel disease, particularly in the distal ileum [14]. Thought to be caused by a complex interplay between genetic susceptibility, environmental factors, disruption to the gut microbiome and mucosal barrier, and an ineffective innate immune system, the condition is characterized by an abnormal T-cell response leading to chronic inflammation [14–16]. This chronic inflammation leads to ulcerations in the intestinal mucosa and development of complications including bowel strictures, fistulas, and abscesses. The disease has a bimodal age of onset that peaks at around ages 20 and 50 [17].

Clinical manifestations of small bowel Crohn’s disease vary, with patients presenting with abdominal pain, diarrhea, weight loss, and anemia, and oftentimes characteristic periods of flares and remissions [18, 19]. Endoscopically, the mucosa may be patchy, with areas of normal mucosa interspersed with areas involved in disease. Mucosa is erythematous with loss of vascularity; development of aphthous ulcers that can eventually form into serpiginous ulcers with areas of edematous mucosa that give the pathognomonic cobblestone appearance [18]. On histology, this small bowel tissue shows patchy chronic inflammatory infiltrate which can be transmural, with non-caseating granulomas [18, 20]. Treatment for Crohn's disease is multidisciplinary and focused on promoting mucosal healing and symptomatic improvement [19]. Medications are centered around immunosuppression to induce and maintain remission, including corticosteroids such as budesonide and prednisone, and biologic agents that can target different functions of the immune response including anti-TNF, anti-integrin, anti-interleukin 12/23, and JAK inhibitors [21]. Surgical treatment of Crohn's disease is indicated in management of disease complications including strictures and fistulas.

### Collagenous Sprue

Similar to collagenous gastritis or collagenous colitis, collagen deposits can form in the small bowel leading to collagenous sprue (CS), which is thought to be the result of triggering a fibrogenic response in the gut with increased collagen production and without adequate fibrinolysis [22, 23]. Collagenous sprue has biopsy appearance of villous blunting with a subepithelial collagen band, usually with an inflammatory component with lymphocytosis in the deposit [24]. CS is closely linked to CeD, although this relationship is not well understood as this has been grouped before with refractory sprue [25]. Patients with positive IgA tissue transglutaminase can have their disease complicated by collagenous sprue, and both CS and CeD carry risks for development of B and T cell lymphomas [25, 26].

CS appears to be mostly in older women, patients will often present with symptoms similar to CeD including chronic diarrhea, weight loss, abdominal pain, but who lack response to a gluten-free diet alone [23, 24]. There is limited data about treatment options, but so far, there has been use of combination of gluten-free diet along with corticosteroids including budesonide with some success [5]. There are several case reports of biologic agents for use of refractory collagenous colitis, but data in proximal small bowel is limited [27, 28].

Additionally, olmesartan can induce a severe sprue-like enteropathy in patients on the medication, which resolves following discontinuation of the medication [29, 30]. A careful medication history can be helpful in the diagnosis and management for these patients.

### Seronegative Celiac Disease

Although this review article is focusing on non-celiac enteropathies, the idea of seronegative CeD should be considered in work-up of enteropathy as it can commonly be missed in absence of celiac antibodies. Seronegative CeD occurs in about 2%− 5% of Celiac diagnoses, making it one of the most common causes of seronegative enteropathies [1, 31]. However we may be underestimating the prevalence of this disease, particularly in non-white populations where antibody response may be less reliable [32]. In contrast to classic CeD, those with seronegative disease have negative transglutaminase (TTG), deamidated gliadin peptide (DGP), and anti-endomysial antibody (EMA) IgA and IgG, while still having HLA testing compatible with development of CeD, villous atrophy on duodenal biopsy, as well as clinical and histologic response to a gluten-free diet [3]. It is hypothesized that this may be secondary to autoantibodies generated in the intestine may not be able to pass into circulation [33]. This can be a challenging disease to diagnose as various other causes of villous atrophy must also be excluded. HLA DQ2 and 8 testing may be helpful as negative results for these haplotypes excludes the possibility of seronegative CeD [3].

The cornerstone for management still revolves around maintaining a gluten-free diet, and since serologic markers cannot be used to assess the response of patients, endoscopic evaluation with duodenal biopsies have to be obtained to assess for healing or resolution [1, 3]. If non-responsive, the diagnosis may need to be reconsidered to assess for the other causes of seronegative enteropathy.

## Infectious

### Tropical

Tropical sprue is seen in individuals who travel to or reside in tropical regions. Characterized by diarrhea, nutrient malabsorption, and weight loss, the etiology of the condition is unknown but presumed to be likely infectious in nature [34]. Histological findings include villous blunting, increased intestinal epithelial lymphocytes, and eosinophilic infiltration of the lamina propria, notably very similar to the histological picture of CeD [7]. Macrocytic anemia due to folic acid or B12 deficiency can be seen after months of disease. Tropical sprue is treated with a 3-to- 6-month course of tetracycline and oral folic acid [35].

### Giardiasis

Giardiasis, an infection by the protozoa Giardia duodenalis, is a leading cause of enteric disease. Spread through contaminated food and water, infection with the parasite can be asymptomatic, but more commonly leads to steatorrhea. Other symptoms of giardiasis include flatulence, abdominal cramping, and bloating, with more chronic presentations associated with weight loss and post-infectious lactose intolerance. Histologically, infection with G. duodenalis leads to lymphocyte mediated microvillous blunting due to parasite-induced chloride hypersecretion and epithelial apoptosis [36]. The infectious mechanism of the protozoa ultimately leads to its malabsorptive presentation. Though self-resolving often within a few weeks, a course of metronidazole is the standard therapeutic intervention [37].

### Whipple’s Disease

Caused by *Tropheryma whippelii*, Whipple’s Disease is a rare systemic condition that causes malabsorptive, watery diarrhea in addition to neurological and cardiac manifestations. Other symptoms of Whipple’s Disease include weight loss, arthralgias, fever, abdominal pain, peripheral edema, pericarditis or endocarditis, frontal release, ataxia, and clonus. Duodenal biopsy revealing PAS-positive macrophages in the lamina propria containing nonacid-fast, Gram-positive bacilli is diagnostic but can be missed if not asked for specifically at biopsy [38]. Treatment typically involves an initial two-week phase of high dose ceftriaxone or penicillin, followed by a one to two yearlong maintenance phase of Trimethoprim-Sulfamethoxazole in order to reduce risk of relapse [39].

### H. Pylori

A Gram-negative, helical bacterium, Helicobacter pylori infects half of the world’s population, though a majority are asymptomatic [40]. Pathogenic infection with a bacterium leads to gastric and duodenal ulcers, chronic gastritis, and increased risk for gastric cancers. If symptomatic, patients may present with dyspepsia, abdominal pain, nausea, and vomiting. Histological examination reveals intraepithelial lymphocytic infiltration of the duodenum which can lead to misdiagnosis of chronic enteropathies [41]. It is therefore recommended to biopsy gastric mucosa when there is concern for enteropathies; alternatively, a breath test or stool antigen can be done if gastric sampling was not done at time of endoscopy. First-line treatment for H. Pylori may vary by region and resistance and local antibiograms should be consulted [42].

### Tuberculosis

Caused by Mycobacterium tuberculosis, tuberculosis (TB) is primarily considered a pulmonary disease. However, extrapulmonary TB accounts for over 20% of reported total TB cases [43, 44]. Abdominal TB, specifically intestinal TB, can lead to villous blunting. Symptoms depend largely on the location and extent of infection but may include diarrhea and abdominal pain, as well as constitutional symptoms such as fever, malaise, night sweats, anorexia, and weight loss. Diagnosis of extrapulmonary TB is more difficult due to low sensitivity and protracted lab result processing [43]. Tissue biopsies containing granulomas and caseation, though not pathognomonic, can support the diagnosis. Mycobacterial culture and/or nucleic acid amplification test (NAAT) can establish diagnosis [45]. Concomitant pulmonary TB, confirmed with positive Tuberculin skin test or IFN-γ releasing assay, may also be supportive in diagnosis of extrapulmonary TB, as extrapulmonary TB is most commonly due to hematogenous spread of pulmonary TB. Medical management includes rifampin, isoniazid, pyrazinamide, ethambutol. Surgical intervention may be warranted for patients with complications such as perforation, abscess, fistula, bleeding, and/or high-grade obstruction.

### Active Viral or Post-Viral

Gastroenteritis is a common cause of illness affecting all ages, though more deadly in the oldest and youngest members of the population. Most causes of gastroenteritis are viral, including rotavirus, norovirus, adenovirus, and astroviruses. Commonly transmitted fecal-orally, these viruses can cause symptoms including watery diarrhea, abdominal pain, nausea, and vomiting that usually improve within 3 days and resolve within 1 week. Histologically, viral gastroenteritis can temporarily cause blunting of villi, shortening of microvilli, dilation of endoplasmic reticulum, and an increase in intracellular multivesicular bodies [46]. Diagnosis is clinical, with history taking that should involve questions regarding sick contacts, travel history, recent antibiotic use, and immunization status. Treatment is supportive, largely focused on rehydration. It should be noted that compared to healthy patients, immunocompromised patients experience a more severe clinical course associated with increased morbidity and mortality due to viral infections [47]. Furthermore, viruses can lead to chronic enteropathies in patients with immunodeficiencies [48, 49].

### HIV

HIV enteropathy is chronic diarrhea without microorganism infection in patients with HIV. Patients experience over a month of diarrhea and unexplained weight loss of at least 10% of their premorbid weight. Villous atrophy, crypt hyperplasia, villous blunting, and lymphocytic infiltrate are seen upon histological evaluation. The mechanism by which this occurs is unclear, but hypotheses include cytokine-mediated destruction and gut-associated lymphoid tissue [50, 51]. The use of highly active antiretroviral therapy (HAART) for patients with HIV enteropathy may benefit some patients, but results in the literature demonstrate a more mixed picture of its efficacy for symptoms [50].

### SIBO

Small intestine bacterial overgrowth (SIBO) is the presence of excess colonic bacteria in the small intestine, leading to symptoms like bloating, flatulence, abdominal discomfort, diarrhea, and abdominal pain [52]. Histologically, duodenal biopsies may show villous blunting and lymphocytic infiltration [53]. SIBO is increased in patients with other primary causes of enteropathy, such as CeD, so overlapping disease should be considered. Diagnosis of SIBO can be made with breath tests that demonstrate elevated hydrogen or methane or with small bowel spiration indicating more than 10^5^ bacteria per milliliter from the small bowel. [52] Due to poor operating characteristics of the breath test and difficulty of obtaining aspirates, empiric treatment in patients with high suspicion of SIBO is reasonable. Rifaximin is the ideal antibiotic therapy due to its targeted nature and efficacy although less expensive antibiotics can be used [52]. The disease can recur over time so patients may require repeated courses of antibiotics.

## Medications

A number of medications can induce enteropathy. Neomycin, for example, may lead to villi height reduction, inflammation, and malabsorption of monoglycerides, fatty acids, cholesterol, and fat-soluble vitamins [26]. Olmesartan can cause a severe enteropathy any time during treatment that resolves with medication cessation, this may extend to a class effect in rare cases [2, 54, 55]. NSAIDs may also lead to drug-induced enteropathy, possibly through reduced mucus and blood flow of intestinal mucosa, mitochondrial damage, and invasion of intestinal cells with pathogens, enzymes, and bile acid [56]. Chemotherapeutic agents like mycophenolate mofetil, methotrexate, and 5-Fluorouracil, and checkpoint inhibitors like pembrolizumab, ipilimumab, and nivolumab may induce histological and morphological changes in the small intestine, but there is significant variation in symptoms and tolerance, dependent on both the individual and the specific chemotherapeutic regimen [55, 57]. Finally, though a rare side effect, severe, refractory enteropathy and colitis can be caused by chimeric antigen receptor T (CAR T) cell therapy, often after completion of CAR T cell therapy [58–60]. A close review of all medications, including over the counter, should be conducted in enteropathy. Repeat biopsies after medication cessation can confirm diagnosis or one can follow for clinical improvement.

## Malignant

### Radiation

Radiation enteropathy can be acute or chronic. While acute radiation enteropathy occurs weeks after treatment, chronic radiation enteropathy may begin months or even years later [61]. Acutely, patients experience nausea, abdominal pain, diarrhea. Chronically, symptoms are due to malabsorption of nutrients and abnormal motility due to mucosal atrophy, vascular sclerosis, and fibrosis. Histologically radiation enteropathy is characterized by leukocytic infiltration, crypt abscesses, ulceration, and sometimes submucosal collagen deposits [62]. It is theorized that disruption of the mucosal barrier leading to subsequent inflammation contributes to the macroscopic and microscopic changes in the intestinal epithelium that leads to symptoms. Management of acute radiation enteropathy is typically symptomatic, utilizing antidiarrheals, antiemetics, and a low fat and lactose free diet may be sufficient to eliminate symptoms. Chronic radiation enteropathy may eventually require surgical intervention, chronic nutrition support and is associated with an increased mortality rate [63].

### Lymphoma

Enteropathy associated T-cell lymphoma (EATL) is a rare type of lymphoma that arises from intestinal epithelial cytotoxic T-cells and is most commonly in the proximal jejunum [64]. Patients can present with symptoms of severe enteropathy as well as obstruction, perforation or bleeding. Endoscopically this can appear as jejunal ulcerations or mass with surrounding inflammation and flattening. Early EATL will have biopsies similar to refractory CeD with severe villous atrophy and lymphocytosis and is associated with Type II refractory CeD. Diagnosis between celiac and EATL can be made by a combination of pathology including flow cytometry and T-cell rearrangement as well as imaging, CT enterography or PET scan. Advanced cases may lead to bowel resection with gross tumor invasion. Appropriate recognition and prompt treatment are key, as the disease is aggressive and has a poor prognosis with median overall survival of 5 to 10 months [65].

### CVID

Common variable immunodeficiency is a primary immunodeficiency which is marked by reduced levels of immunoglobulins and resultant impaired antibody response [66]. CVID patients have a high susceptibility to recurrent bacterial infections of the gastrointestinal and respiratory tracts. Autoimmune inflammatory and malignant complications driven by the underlying immune dysregulation are common in this patient population. It is estimated that 9 to 17% of patients with CVID will suffer from enteropathy with associated increased mortality. [67–69] These patients often present with severe diarrhea and malabsorption leading to a need for parenteral nutrition. CVID patients with GI complications are more likely to have comorbid conditions such as liver and lung involvement, granulomatous disease, lymphoma/hematologic complications and rheumatologic disorders than those without GI symptoms [70]. Clinical suspicion for NCE should be high in any patient with CVID who presents with diarrhea, weight loss or micronutrient deficiencies. In addition to villous atrophy, histology may show lack of plasma cells (68%), prominent lymphoid aggregates (47%), increase apoptosis (20%), increased intraepithelial lymphocytes (63%) and granulomas (11%) [71]. Treatment often includes immunoglobulin therapy which often does not improve outcomes, corticosteroids, and nutrition support. There are case reports of biologic therapy (ie infliximab, vedolizumab) showing positive outcomes, but this has yet to be validated in larger trials [72–78].

## Eosinophilic

Eosinophils are a normal part of the small intestine (up to 52 eos/hpf in the duodenum, and 56 eos/hpf in ileum) but can be elevated in certain pathology already discussed in this review (ie infections, celiac, crohn’s). Primary eosinophilic enteritis (EoN) has a prevalence of 2.1 to 3.1 per 100,000 but may be under-identified due to lack of awareness/appropriate sampling [79–81]. Similar to other eosinophilic diseases of the GI tract, EoN is commonly associated with allergic disorders such as allergic rhinitis, asthma, food and drug allergies. The primary mechanism of inflammation is a Th2 driven response with elevated IgE, type 2 cytokines and increased peripheral blood eosinophils [82]. Dietary elimination are effective treatments in some patients with EoN with cows milk as the most common trigger [83]. Pharmacologic treatments for EoN are mostly case reports and mirror treatment of eosinophilic esophagitis including oral and topical corticosteroids (budesonide), mast cell stabilizers and leukotriene inhibitors, and biologic or other immunosuppressants [84]. Dupilumab, an FDA approved medication for EoE, is currently in clinical trials for EoN.

### Idiopathic

In some cases the cause of enteropathy will not be identified. In one center's experience of seronegative enteropathy over 15 years, 36/200 cases were deemed idiopathic. The majority of these, however, resolved after 9 months with no treatment [4]. In settings of severe disease, empiric treatment may be considered with close monitoring for clinical and histologic improvement.

## Conclusion

Although CeD is by far the most common cause of enteropathy, it is important to distinguish when small bowel disease is not from celiac and to identify appropriate etiology and treatment. This can be difficult in settings of low IgA, gluten restriction prior to diagnosis or inadequate small bowel sampling. Thorough review of a patients’ detailed history, medications and lab results may lead to diagnosis and tailor treatment choices. Direct discussion with an expert pathologist is recommended in difficult cases. Supportive nutrition care should be initiated early when applicable to minimize morbidity. Empiric treatment with budesonide can be considered in situations where diagnosis is not clear [3].

## Key References

- 10.1016/j.mayocp.2017.10.025
  - ⚬ A broad review that highlights the importance of differentials for NCEs. The article provides valuable insights into the diagnostic challenges and the need for careful clinical evaluation to avoid misdiagnosis.
- 10.1053/j.gastro.2020.08.061
  - ⚬ Provides guidelines for diagnosis and management for NCEs, offering a comprehensive approach to these complex conditions. The article aids in navigation of the diagnostic challenges of seronegative enteropathies and further emphasizes the detailed history taking and a personalized approach to care.
- 10.1111/his.14262
  - ⚬ This reference emphasizes how tissue biopsy and histological analysis provide valuable insights into the extent of intestinal damage in patients with NCEs and how histological findings may help guide effective treatment strategies.

## Data Availability

No datasets were generated or analysed during the current study.
